# Impact of Fermentation Conditions on Physicochemical Properties, Antioxidant Activity, and Sensory Properties of Apple–Tomato Pulp

**DOI:** 10.3390/molecules28114363

**Published:** 2023-05-26

**Authors:** Jing Yuan, Haiyan Zhang, Chaozhen Zeng, Juan Song, Yuwen Mu, Sanjiang Kang

**Affiliations:** Agricultural Product Storge and Processing Research Institute, Gansu Academy of Agricultural Sciences, Lanzhou 730070, China; yuanjing@gsagr.cn (J.Y.); zh_hy208@163.com (H.Z.); zengchaozhen@gsagr.cn (C.Z.); songjuan@gsagr.cn (J.S.); muyuwen-910@163.com (Y.M.)

**Keywords:** fermentation, apple–tomato pulp, physicochemical properties, polyphenols, flavonoids, antioxidant activity, flavour, sensory properties

## Abstract

The aim of the study was to optimize the conditions [inoculum size (4, 6, and 8%), fermentation temperature (31, 34, and 37 °C), and apple: tomato ratio (2:1, 1:1, and 1:2)] on the viable cell count and sensory evaluation in apple–tomato pulp by response surface methodology (RSM), and determine the physicochemical properties, antioxidant activity, and sensory properties during fermentation. The optimal treatment parameters obtained were an inoculum size of 6.5%, a temperature of 34.5 °C, and an apple: tomato ratio of 1:1. After fermentation, the viable cell count reached 9.02 lg(CFU/mL), and the sensory evaluation score was 32.50. During the fermentation period, the pH value, total sugar, and reducing sugar decreased by 16.67%, 17.15%, and 36.05%, respectively. However, the total titratable acid (TTA), viable cell count, total phenol content (TPC), and total flavone content (TFC) increased significantly by 13.64%, 9.04%, 21.28%, and 22.22%, respectively. The antioxidant activity [2,2-diphenyl-1-picrylhydrazyl (DPPH) free-radical scavenging ability, 2,2′-azino-di(2-ethyl-benzthiazoline-sulfonic acid-6) ammonium salt (ABTS) free-radical scavenging ability, and ferric-reducing antioxidant capacity power (FRAP)] also increased by 40.91%, 22.60%, and 3.65%, respectively, during fermentation. A total of 55 volatile flavour compounds were detected using HS-SPME-GC–MS among the uninoculated samples and fermented samples before and after fermentation. The results showed that fermentation increased the types and total amount of volatile components in apple–tomato pulp, and eight new alcohols and seven new esters were formed. Alcohols, esters, and acids were the main volatile components in apple–tomato pulp, accounting for 57.39%, 10.27%, and 7.40% of the total volatile substances, respectively.

## 1. Introduction

The rise of health-conscious consumers had resulted in acceptance towards nutritious and functional foods [1]. Based on this, many processing methods and advanced technologies to improve the nutrition and functionality of food products are constantly being explored. Among the methods that can be used to improve the nutritional value of food, fermentation is one of the most promising [2].

Probiotics are defined as “a class of micro-organisms that benefit the host’s health” [3]. Several studies have shown that probiotics have antibacterial and anti-inflammatory properties [4] and anticancer properties [5], reduce serum cholesterol [6], regulate intestinal flora, and enhance the function of the intestinal barrier [7]. Health benefits of specific probiotic bacterial strains such as *Lactobacillus*, *Bifidobacterium*, *Escherichia*, *Enterococcus*, *Streptococcus*, *Leuconostoc*, *Pediococcus*, and *Bacillus* have predominantly been reported [8,9,10,11,12]. Currently, people obtain probiotics mainly from fermented dairy products. However, Silanikove found that approximately 75% of people worldwide have lactose intolerance and milk protein allergies, and dairy products also have a high cholesterol content, which greatly limits people’s access to probiotics [13]. Therefore, it is necessary to combine the benefits of fermented plant-based substrates and probiotics [14]. As a result, new food matrices as probiotic carriers have been tested. Fruits and vegetables (F&V) are rich in dietary fiber, vitamin C, vitamin B_2_, carotene, pectin, and organic acids. Several studies have shown that F&V are great probiotic sources because of their rich nutrients [13,15,16,17]. Thus, fermented F&V is a promising strategy to enhance bioactive compounds and their activity properties [18,19,20,21].

Apples and tomatoes have high nutritional quality, and various studies have shown that their juices are suitable raw materials for fermented beverages [22,23,24]. Apples contain carbohydrates, polyphenols, organic acids, vitamins, and minerals [25]. Among these, polyphenols have strong antioxidant and free-radical scavenging abilities. It prevents chronic diseases such as lung cancer, asthma, and cardiovascular diseases [26,27]. Tomatoes are rich in vitamin C, lycopene, soluble sugars, and organic acids, which have antioxidation functions, improving human resistance to diseases and cancers [28].

Several parameters influence the fermentation ability of lactic acid bacteria (LAB), such as pH, inoculum size, fermentation temperature, and fermentation time [29]. The final quality of the product depends largely on the fermentation conditions. Therefore, when developing functional probiotic foods, it makes sense to carefully study the interaction between probiotics and food ingredients. According to Liao et al., the modeling and optimization results show that the mixed *L. fermentum* and *L. plantarum* at 0.5:0.5, can maximize the total phenolic content in fermented blueberry juice. After fermentation, total phenolic, ferulic acid, rutin, and quercetin-3-rhamnoside contents were 82.19%, 15.22%, 79.08%, and 98.59% higher than the unfermented juice [30]. Yuan et al. investigated the fermentation process conditions and quality of green jujube wine. The results show that the optimal process conditions were initial sugar of 24%, yeast addition of 0.3%, fermentation time of 8 d, and SO_2_ treatment of 80 mg/L. The chemical composition and antioxidant capacity of the optimized samples were evaluated. The results showed that jujube wine had a high content of antioxidant substances and good biological activity. After fermentation, the content of aldehydes, ketones, heterocycles, and aromatic compounds were significantly reduced, and the production of esters and alcohols [31].

The purpose of this research was to evaluate the conditions on the viable cell count and sensory evaluation in apple–tomato pulp by response surface methodology (RSM). Moreover, the changes during fermentation in physicochemical indices, antioxidant capacity, and volatile compounds were also studied.

## 2. Results and Discussion

### 2.1. Optimization and Analysis

#### 2.1.1. Response Surface Methodology (RSM) Model

Based on preliminary experiments, the optimal levels of the significant factors (inoculum size, fermentation temperature, and apple: tomato ratio) and interaction effects on the viable cell count and sensory evaluation of fermented apple–tomato pulp were explored by the BBD design of RSM (Table 1), and the variance analysis of the regression RSM model is given in Table 2. As shown in Table 2, the effect of three independent variables on the viable cell count and sensory evaluation were found to be extremely significant (*p* < 0.0001), hence its ability to elucidate the real relationship between the responses and the factors. The lack of fit with *p* values was not significant (*p* > 0.05), demonstrating that the model fits with the data. Good coefficients of determination were also obtained (R^2^ = 0.9847 and R^2^ = 0.9910, respectively). It shows that the model fits well between the factors and the response values, and the established regression model reflects that the real relationship between the response and independent variables was a reliable regression model.

The viable cell count (*Y*_1_) and sensory evaluation (*Y*_2_) regression equations were obtained after multiple regression fittings of the test results (Table 2). The regression model equations of *Y*_1_ and *Y*_2_ were calculated as follows in Equations (1) and (2):

*Y*_1_ (Viable cell count, lg(CFU/mL)) = 8.98 + 0.27*X*_1_ + 0.36*X*_2_ + 0.11*X*_3_ + 0.18*X*_1_*X*_2_ − 0.003*X*_1_*X*_3_ + 0.18*X*_2_*X*_3_ − 0.23*X*_1_^2^ − 0.38*X*_2_^2^ − 0.12*X*_3_^2^ (1)

*Y*_2_ (Sensory evaluation, scores) = 34.05 + 0.31*X*_1_ − 0.29*X*_2_ − 0.63*X*_3_ − 0.81*X*_1_*X*_2_ − 0.47*X*_1_*X*_3_ − 0.14*X*_2_*X*_3_ − 2.26*X*_1_^2^ − 3.14*X*_2_^2^ − 1.43*X*_3_^2^ (2)

*X*_1_ (inoculum size), *X*_2_ (temperature), and *X*_3_ (apple: tomato ratio) were factor horizontal coding values, and *Y* is the fuzzy evaluation value.

**Figure 1:**
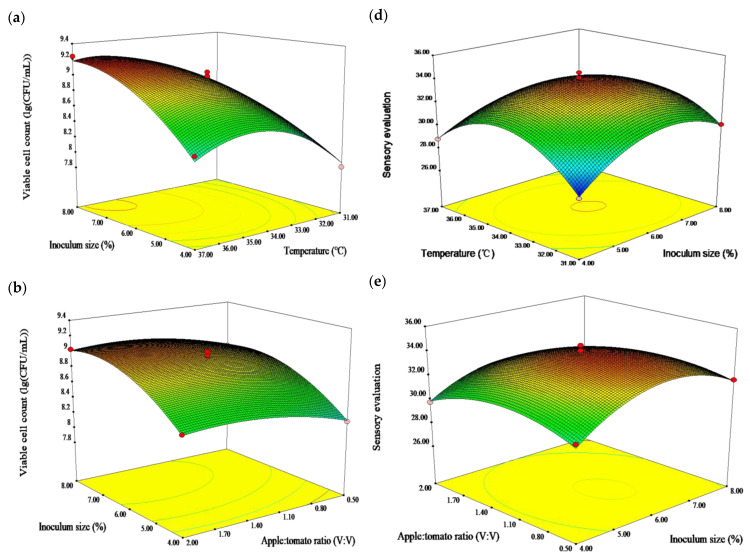

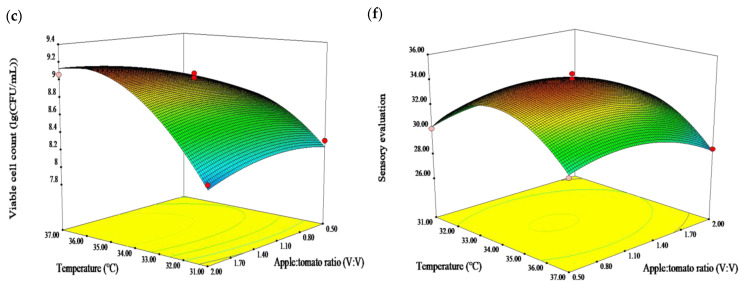
Response surface plot showing the effect of independent variables on viable cell count and sensory evaluation [(**a**) inoculum size and temperature; (**b**) inoculum size and apple: tomato ratio; (**c**) temperature and apple: tomato ratio on viable cell count of fermented apple-tomato pulp; (**d**) temperature and inoculum size; (**e**) apple: tomato ratio and inoculum size; (**f**) temperature and apple: tomato ratio on sensory evaluation of fermented apple-tomato pulp].

Figure 1a–c shows three-dimensional response surface plots describing the interaction effect of the two factors. The viable cell count regression model analysis indicated that the model was significant (*p* < 0.01), and the misfit item was not significant (*p* > 0.05). The coefficient of determination R^2^ and the adjusted R^2^ were 0.9847 and 0.9650, respectively. The *p* values indicated that the impact of inoculum size (*X*_1_), temperature (*X*_2_), apple: tomato ratio (*X*_3_), interactions *X*_1_*X*_2_ and *X*_2_*X*_3_, and the quadratic terms *X*_1_^2^ and *X*_2_^2^ on the viable cell count were extremely significant (*p* < 0.01), and the quadratic term *X*_3_^2^ had a marked effect (*p* < 0.05). The interaction *X*_1_*X*_3_ was inessential (*p* > 0.05). F values revealed the effects of the following factors: temperature > inoculum size > apple: tomato ratio.

Temperature was found to have the main effect on the viable cell count (Figure 1a–c), suggesting that the viable cell count was increased before 34.5 °C and decreased slightly afterwards. The inoculum size tended to increase the release of the viable cell count. The viable bacteria count reached a maximum (9.10 lg(CFU/mL)), when the apple: tomato ratio was 1:1 and decreased afterwards. This is in line with the finding of Ren et al. that temperature and inoculum size had a positive effect on the viable cell count [32].

The sensory evaluation of fermented apple–tomato pulp ranged from 27.68 to 33.98, depending on the inoculum size, fermentation temperature, apple: tomato ratio, and their interaction (Figure 1d–f). The sensory evaluation score increased with the increase of the apple: tomato ratio, inoculum size, and temperature, reaching a maximum (33.85) at an inoculum size of 6.5%, apple: tomato ratio of 1:1, and temperature of 34.5 °C, and then decreased.

The sensory evaluation regression model analysis indicated that the model was significant (*p* < 0.01), and the misfit item was not significant (*p* > 0.05). The coefficient of determination R^2^ and the adjusted R^2^ were 0.9910 and 0.9795, respectively. The *p* values indicated that the impact of the apple: tomato ratio (*X*_3_), interaction *X*_1_*X*_2_, and the quadratic terms *X*_1_^2^, *X*_2_^2^, and *X*_3_^2^ on sensory evaluation were extremely significant (*p* < 0.01), and the inoculum size (*X*_1_), fermentation temperature (*X*_2_), and interaction *X*_1_*X*_3_ had marked effects (*p* < 0.05). The interaction *X*_2_*X*_3_ was inessential (*p* > 0.05). Meanwhile, the effect of the experimental factors on the sensory evaluation was apple: tomato ratio > inoculum size > temperature.

#### 2.1.2. Determination and Verification of Fermentation Process Parameters

Under the optimization process of Design-Expert V8.0.6, the optimal fermentation parameters for LAB-fermented apple–tomato pulp were found to be an inoculum size of 6.5%, a temperature of 34.5 °C, and an apple–tomato ratio of 1:1. Under these conditions, the viable cell count of fermented apple–tomato pulp was 9.10 lg(CFU/mL), and the sensory evaluation score was 33.85. Afterwards, three parallel tests yielded a viable cell count of 9.02 lg(CFU/mL) and sensory evaluation score of 32.50. The results show that the predicted values of each index were in good agreement with the experimental values (*p <* 0.05).

**Figure 2:**
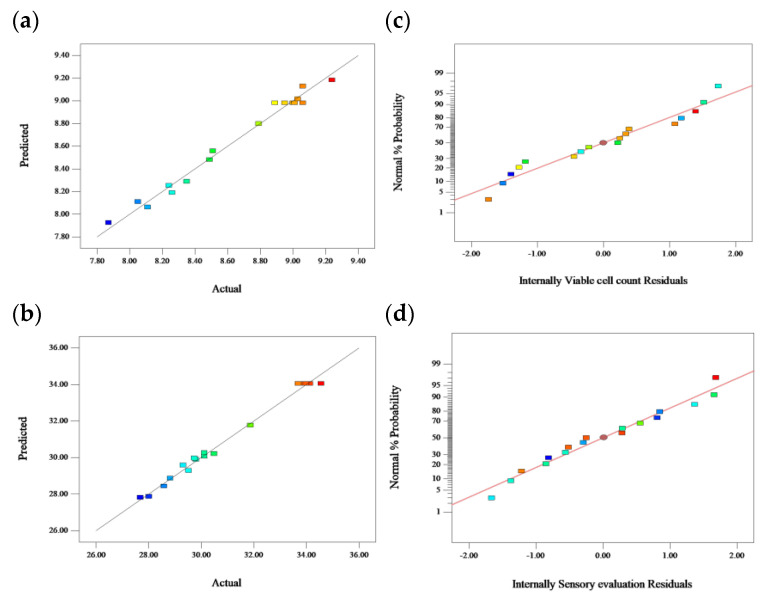
Comparison between predicted and actual values [(**a**) viable cell count(lg(CFU/mL)); and (**b**) sensory evaluation(scores)] and normal % probability and internally residuals [(**c**) viable cell count(lg(CFU/mL)); and (**d**) sensory evaluation(scores)].

The C.V. values of viable cell count and sensory evaluation score were 0.93 and 1.09, respectively. These results demonstrated the reliability and reproducibility of the experiment. In addition, the diagnostic plot (Figure 2) depicts a high correlation between the predicted and the experimental values.

### 2.2. Changes in the Physicochemical Properties of Fermented Apple–Tomato Pulp

#### 2.2.1. Viable Cell Count, pH, and Total Titratable Acid (TTA)

**Figure 3:**
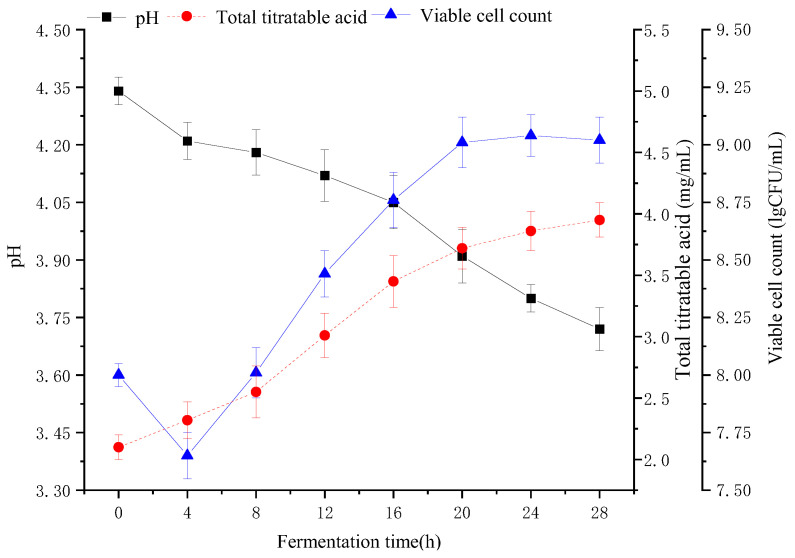
Changes in viable cell count, pH, and TTA by fermentation for 28 h.

The viable bacteria showed a trend of first decreasing and then increasing (Figure 3). In the early stage of fermentation, LAB needed to adapt to the F&V pulp environment, and the viable count decreased to 7.65 lg(CFU/mL)at the fourth hour. The difference between the culture medium and F&V fermentation substrate resulted in a decline in the growth rate in the earlier fermentation period [33]. Subsequently, the viable bacteria increased gradually and reached a maximum of 9.04 lg(CFU/mL)at 24 h of fermentation. Pereira et al. and Fonteles et al. evaluated the growth conditions of *L. casei* fermentation in cashew apple and cantaloupe juices, respectively. Similar conclusions were reached [4,34]. Organic acids can promote digestion and soften blood vessels. The content of organic acids affected the stability, sensory quality, and nutritional quality of apple–tomato pulp. With the extension of fermentation time, the accumulation of TTA in the fermentation system increased gradually and reached 3.95 mg/mL at 28 h. Zhang et al. examined the TTA of fermented elderberry juice during fermentation and obtained 3.5 mg/mL, and a similar result (3.72 mg/mL) was obtained at 20 h [35]. The pH dropped along with the fermentation time due to the production of organic acids, such as lactic acid, acetic acid, and malic acid. Lower pH inhibits the growth of pathogens and many spoliating micro-organisms [36].

#### 2.2.2. Total Sugar and Reducing Sugar

**Figure 4:**
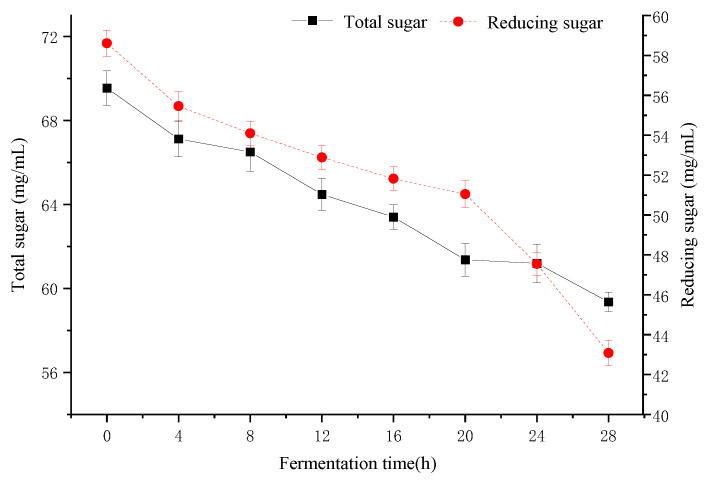
Changes in total sugar and reducing sugar by fermentation for 28 h.

Figure 4 shows the carbohydrate consumption during fermentation. Sugars are the main carbon source for microbial growth [37]. At the beginning of fermentation, the total sugar and reducing sugar contents were 69.54 ± 0.83 mg/mL and 58.61 ± 0.66 mg/mL, respectively. With the extension of fermentation time, the total sugar and reducing sugar showed a decreasing trend, and the total sugar tended to flatten out at 20 h (61.36 ± 0.78 mg/mL), while the reducing sugar decreased by 36.05% at 28 h. Wang et al. reported that reducing sugar was a very good carbon and energy source for *lactobacilli* and *bifidobacteria* [38]. Minervini and Calasso found that *L. paracasei* CASEI 431 consumes reducing sugars and produces lactic acid during fermentation [39]. Our result had the same point of view.

### 2.3. Total Phenolic Content, Total Flavonoid Content, and Antioxidant Capacity Analysis

**Figure 5:**
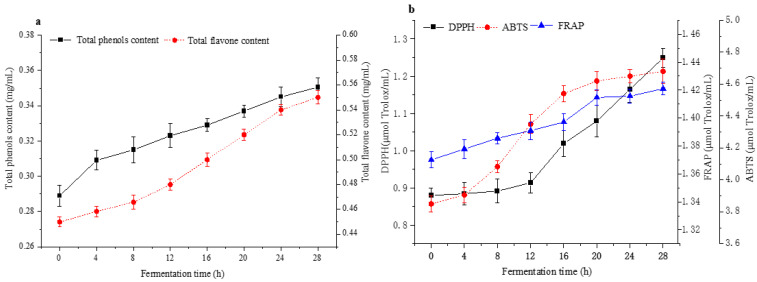
Changes in total phenols content (TPC), total flavone content (TFC), antioxidant capacity by fermentation for 28 h [(**a**) Changes in the content of TPC and TFC during fermentation; (**b**) Changes in the content of DPPH, ABTS and FRAP during fermentation].

The total phenol content (TPC) and total flavone content (TFC) are crucial functional substances in F&V, which are beneficial to anti-inflammatory, antioxidant, and anticancer activities [1,40]. TPC, TFC, DPPH, FRAP, and ABTS are presented in Figure 5. The TPC and TFC in apple–tomato pulp were significantly increased after fermentation (Figure 5a. Furthermore, they increased by 21.28% and 22.22% after 28 h, respectively. This result could be attributed to the released aglycones produced by microbial enzymes [41]. The view was consistent with the conclusion of Qi et al., who fermented Chinese wolfberry juice by LAB [42]. 

Three antioxidant indicators, including DPPH scavenging ability, ABTS scavenging ability, and ferric-reducing antioxidant power (FRAP), were used to evaluate the antioxidant activities of apple–tomato fermentation pulp. Figure 5b shows changes in the antioxidant capacity of the sample during the fermentation process. The DPPH scavenging ability had the same trend as the ABTS scavenging ability, and they both increased by 40.91% and 22.60% compared with before fermentation, respectively. The change in DPPH scavenging ability and ABTS scavenging ability was primarily related to the change in polyphenol content [31]. This may be because of the enzyme-induced and nonenzymatic oxidation mechanism of *L. plantarum* during fermentation, which enhanced the content of active small molecules with antioxidant capacity in the fermentation system, thus improving the antioxidant capacity of the system [43]. On the other hand, FRAP scavenging ability did not change significantly during fermentation (increased 3.65%), which was consistent with Pereira et al.’s conclusions [4].

The polyphenols, flavonoids, and other substances contained in the fermented F&V juice could effectively scavenge free radicals [44,45]. Therefore, a correlation analysis between antioxidant capacities and polyphenols and flavonoids was further conducted. The TPC, TFC, and antioxidant activity of fermented apple–tomato pulp were altered with fermentation time, and the association of antioxidant activity with TPC and TFC was assessed by linear regression. The corrected Pearson coefficient *R* was applied, and the data are depicted in Table 3.

The TPC in the fermentation system was positively correlated with DPPH, FRAP, and ABTS (Table 3). TPC showed a highly significant (*p* < 0.05) correlation with TFC, DPPH, FRAP, and ABTS (*r* = 0.96, *r* = 0.90, *r* = 0.97, and *r* = 0.95, respectively). TFC (*p* < 0.05) was correlated with DPPH, FRAP, and ABTS (*r* = 0.98, *r* = 0.98, and *r* = 0.94, respectively). This result is supported by the literature on Fuji apple pulp (Pearson’s *r* ≥ 0.97) [46]. During the early stage of fermentation, the binding of polyphenols (flavonoids) with pectin, cellulose, arabinoxylan, sugar, and structural proteins is disrupted by microbes [47], which leads to the release of bound phenolics [48]. 

### 2.4. HS-SPME/GC–MS Analysis

The changes in volatile compounds before and after fermentation in apple–tomato pulp were analyzed by HS-SPME/GC–MS. In the fermentation process, a total of 55 kinds of volatile compounds were detected, including 19 alcohols, 11 esters, 6 acids, 8 ketones, 2 aldehydes, 3 phenols, and 6 other kinds (Table 4).

After LAB fermentation, more than 16 kinds of volatile flavour substances increased, and the total amount increased by 161.74%. The newly generated volatile substances were mainly alcohols. Alcohols, esters, and acids were the main volatile components in apple–tomato pulp, accounting for 57.39%, 10.27%, and 7.40% of the total volatile substances, respectively. In this study, the main alcohol detected in the fermented pulp was 1-hexanol, and a high concentration of 1-hexanol (189.58 μg/L) endows apple–tomato pulp with citrus and apple flavours. There were eight new alcohols, including 1-hexanol, (E)-2-hexen-1-ol, (Z)-3-hexen-1-ol, geraniol, 2-methyl-3-octanol, 1-heptanol, 1-octanol, and 1-dodecanol, endowing sweet notes of rose, grass, and clove. When LAB metabolize lactose, amino acids, methyl ketones, and so on, the corresponding aldehydes can be reduced to alcohols by dehydrogenase [43]. The content of esters (81.71 μg/L) was second only to alcohols. Esters contributed greatly to the aroma of apple–tomato pulp. The new esters produced by lactic acid fermentation are mainly 2-hexen-1-ol-acetate, acetic acid-butyl ester, methyl butyrate, and dibutyl phthalate, and they have the aromatic smell of pineapple, banana, and apple, and contribute more to the aroma. This may be due to the catalysis of the complex enzyme system of LAB, which produces certain esters from alcohols and organic acids [48]. Previous studies have also shown that the main contributors to aroma were ethyl acetate, 2-hexen-1-ol-acetate, methyl butyrate, dibutyl phthalate, and so on [29].

Compared with unfermented apple–tomato pulp, the fermentation of LAB increased the total amount of acids by 11 times, mainly acetic acid, which was mainly produced by LAB through the pyruvate–formic acid cleavage pathway. In addition, 2,3-dihydro-benzofuran and 4-vinylphenol were produced, and 4-vinylphenol had an unpleasant smell of tobacco, which had an adverse effect on the aroma.

**Figure 6:**
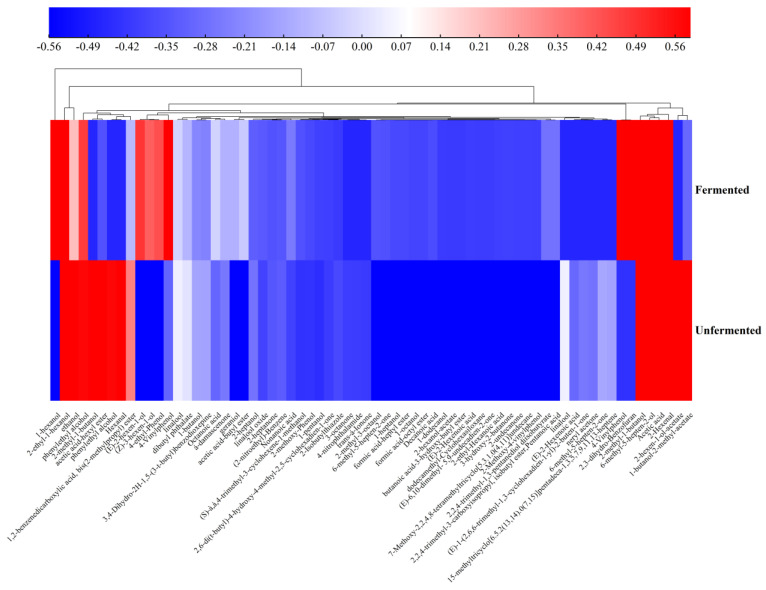
Identification of the volatile flavor components of apple–tomato pulp samples.

As shown in Figure 6, the heatmap and cluster analysis of volatile substances in unfermented and fermented apple–tomato pulp were evaluated. The results show that fermentation increased the types and total amount of volatile components in apple–tomato pulp and could significantly change the abundance of volatile metabolites.

## 3. Materials and Methods

### 3.1. Materials

Fuji apples (Nagafu No. 2) were obtained from JingNing County (Pingliang, China), and tomatoes were purchased from a local supermarket (Hualian Supermarket, Lanzhou, China). Two commercial strains, *Bifidobacterium adolensentis* CICC 6175 and *Lactiplantibacillus pentosus* CICC 24202, were provided by the China Center of Industrial Culture Collection (CICC, Beijing, China). CICC 6175 is a probiotic, and CICC 24202 can produce γ-aminobutyric acid (GABA), which has a prebiotic function. MRS (Modified deMan, Rogosa, and Sharpe) culturing media were acquired from Aobox Co. (Beijing, China). All analytical- or high-performance liquid chromatography (HPLC)-grade chemical and biochemical reagents were provided by Sinopharm Chemical Reagent Co., Ltd. (Shanghai, China) or Sigma Aldrich (Beijing, China).

### 3.2. Apple–Tomato Pulp Preparation

First, apples and tomatoes were rinsed, then the apples were enucleated, and their juice was extracted by a fruit juicer (Joyoung, China). Tomatoes were removed from the stalks, cut into pieces, and beaten (Scientz, China) to obtain their pulp. Apple juice and tomato pulp were mixed in different proportions in 250 mL conical flasks and heated in a water bath at 85 °C for 15 min.

### 3.3. Fermentation Experiments

The strains were incubated with 100 mL of MRS broth at 37 °C for 48 h in anaerobic conditions for primary cultures. The primary culture (1 mL) was inoculated with 150 mL of MRS broth for 24 h at 37 °C under anaerobic conditions for the secondary cultures. Then, the activated cultures were centrifuged at 4000× *g* for 10 min at 4 °C (Cence H1850R benchtop centrifuge, CENCE, Hunan, China). The pellets were washed with stroke-physiological saline solution (0.9% (*w*/*v*)) twice and then resuspended in 1 mL stroke-physiological saline solution to obtain ~10^8^ CFU/mL immediately before substrate inoculation.

For fermentation, each concentration of apple–tomato pulp (200 mL) was mixed with the bacterial cultures to prepare an 8 log CFU/mL sample (*B. adolensentis*: *L. pentosus* was 1:1, *v*:*v*). After that, the inoculated apple–tomato pulp was incubated at 34 °C in the dark for 24 h statistically. The samples were kept at 4 °C until subsequent analyses.

### 3.4. Experimental Design

The effects of inoculum size, fermentation temperature, and apple: tomato ratio treatments on the quality of fermented apple–tomato pulp were preliminarily investigated by using single-factor experiments. The factors chosen were inoculum size (4, 6, 8, 10, and 12%), fermentation temperature (28, 31, 34, 37, and 40 °C), and apple–tomato ratio (3:1, 2:1, 1:1, 1:2, and 1:3). which played a significant effect on the pulp quality (based on previous study, and unpublished data).

A three-factor BBD was employed to study the effect of probiotic fermentation parameters on the viable cell count and sensory evaluation of the apple–tomato pulp. As shown in Table 1, the independent variables applied in the experimental design were the inoculum size (6, 8, and 10%), fermentation temperature (31, 34, and 37 °C), and apple–tomato ratio (2:1, 1:1, and 1:2). The response factors were viable cell count and sensory evaluation.

The polynomial regression equation described the second-order response as a function of the experiments. The quadratic polynomial model fitted to each response value was as follows in Equation (3):

(3) $$Y=\beta_{0}+\sum_{i=1}^{3}\beta_{i}X_{i}+\sum_{i=1}^{3}\beta_{ii}X_{i}^{2}+\sum \sum_{j=i+1}^{3}\beta_{ij}X_{i}X_{j}$$

where *Y* is the response value; $\beta_{0}$, $\beta_{i}$, $\beta_{ii}$, and $\beta_{ij}$ are the regression coefficients for the intercept, linearity, quadratic, and the interaction of the model; and $X_{i}$ and $X_{j}$ are the independent variables.

### 3.5. Sensory Analysis

The sensory evaluation of fermented apple–tomato pulp was evaluated by a 9-scale method [49]. The assessment team consisted of 9 sensory-trained students, and mouthwash was provided to raters between the evaluations of different samples to avoid lingering aftertaste. The color (0–9), smell (0–9), taste (0–9), and acceptability (0–9) were evaluated. The means of scales were: 1 = dislike extremely; 2 = dislike very much; 3 = dislike moderately; 4 = dislike slightly; 5 = neither like nor dislike; 6 = like slightly; 7 = like moderately; 8 = like very much; and 9 = like extremely.

### 3.6. Analysis of Nutrients

#### 3.6.1. Viable Cell Count, pH, and Total Titratable acid (TTA)

The viable cell count was assessed via the pouring plate counting protoco [50]. The results are expressed as the logarithm of living bacteria in the fermentation broth (Lg colony-forming units/mL) in lg (CFU/mL) units. The pH of the pulp was determined by a precision pH meter (PB-10, Sartorius (Shanghai) Trading Co., Ltd., Shanghai, China). The TTA was measured by the acid–base titration method and was calculated based on the conversion coefficient of lactic acid [51].

#### 3.6.2. Total Sugar and Reducing Sugar

High-performance liquid chromatography (HPLC) analysis was used to detect and quantify total sugar and reducing sugar (2695, Waters Corp., Wilmington, MA, USA). The sample was diluted with ultrapure water until the sugar content was approximately 5 g/L and filtered with a 0.45 μL water system filtration membrane. Twenty microliters of sample were injected onto a chromatograph equipped with a Sugar-Pak 1 column (300 × 6.5 mm) and a refractive index detector. Acetonitrile: water (75:25) was used as the mobile phase, and the flow rate was 0.5 mL/min. The column temperature was maintained at 90 °C and that of the detector at 45 °C [52]. The reducing sugar was tested by the dinitrosalicylic acid colorimetry (DNS) method [53].

### 3.7. Antioxidant Capacity

#### 3.7.1. Determination of Total Phenolic Content (TPC)

The TPC was assessed by a SPECTR Amax 190 microplate reader (Molecular Devices Corp., Sunnyvale, CA, USA) via the Folin-phenol colorimetric method [54] as described by Yang and Sun with slight optimizations [22]. TPC was the equivalent of gallic acid in each milliliter of the sample, abbreviated as mg/mL.

#### 3.7.2. Determination of Total Flavonoid Content (TFC)

The TFC was determined using a NaNO_2_-Al(NO_3_)_3_ method [55]. A 2 mL sample was mixed with 2 mL of 70% ethanol and 0.75 mL of 5% NaNO_2_ and incubated at 25 °C for 6 min. Then, 0.5 mL of 10% Al(NO_3_)_3_ and 4 mL of 5% NaOH were added, and the absorbance was measured at 510 nm. The TFC was calculated from a standard calibration curve of rutin, abbreviated as mg/mL.

#### 3.7.3. DPPH· Radical Scavenging Activity

The DPPH· free radical method was carried out according to Loganayaki et al. with modifications [56]. Twenty microliters of the supernatant were added to 380 μL of DPPH· solution (0.1 mM). The tubes were allowed to stand for 20 min at 27 °C. Changes in the absorbance of the samples were measured at 517 nm. The antioxidant efficiency was determined as the time when the concentration of substrate caused a 50% loss in absorbance, and the results were depicted as Trolox equivalents.

#### 3.7.4. ABTS Free-Radical Scavenging Assay

This assay was carried out by slightly optimizing a previously reported method [57]. Briefly, 3.3 mg of sodium persulfate and 19.4 mg of ABTS·^+^ were added to an aluminum-wrapped amber with 5 mL of distilled water, mixed well, and left for 16 h in the dark. The reagents were prepared by mixing anhydrous ethanol 1:10 dilution, 190 mL diluted ABTS·^+^, and 10 mL sample for 20 min. The absorbance was taken by a SPECTRA max 190 microplate reader at 734 nm, and the results were depicted as Trolox equivalents.

#### 3.7.5. Ferric-Ion-Reducing Antioxidant Power (FRAP)

The FRAP analysis was conducted by slightly optimizing a previously reported method [58]. The reagent comprised 25 mL of acetate buffer solution (0.3 M, pH 3.6), 2.5 mL of TPTZ (0.01 M) in 40 mmol/L HCl, and 2.5 mL of FeCl_3_ (0.02 M), which were mixed, shaken, and warmed at 37 °C for 30 min. Then, 190 mL of these reagents was added to 10 mL of the sample at 37 °C for 20 min, and its absorbance was measured by a SPECTRA max 190 microplate reader at 593 nm. One milliliter of the sample produced Fe^2+^-TPTZ/min, a unit of enzyme activity. The total antioxidant capacity was depicted as Trolox equivalents.

### 3.8. Composition of Volatiles

Headspace solid phase microextraction/gas chromatography-mass spectrometry (HS-SPME/GC–MS) (GC:TRACE 1310, MS:ISQ-LT, Thermo Fisher Scientific Co., Ltd., Waltham, MA, USA) was utilized to determine the volatile profiles with and without fermentation. One gram of sodium chloride and 50 μL of 3-octanol were added to 5 mL samples, mixed into 15 mL headspace bottles, and incubated overnight at 4 °C. Then, the sample was placed into the TriPlus RSH Autosampler-SPME system for extraction, adsorbed at 60 °C for 30 min, and held for 5 min.

One milliliter of the above mixture was added to 20 mL headspace injection vials. Volatile substances were isolated using a DB-WAX chromatographic column (30 m × 0.25 mm × 0.25 μm). Gas chromatography (GC) spectrometry parameters were set as follows: the injection temperature was 25 °C, and He served as the carrier gas at a flow rate of 1.2 mL/min. The injection volume was 1 μL, with split injection (40:1). The temperature was kept at 40 °C for 3 min, then increased to 180 °C at a rate of 6 °C/min, held for 2 min, and increased to 230 °C at a rate of 10 °C/min, held for 6 min.

The mass spectrometry parameters were set as follows: electron bombardment ion source, ionization energy was set as 70 eV, ion source temperature was 200 °C, interface temperature was 250 °C, and scanning range was 33.00~450.00 amu.

### 3.9. Data Analysis

All methods were carried out three times. SPSS 16.0 was used to assess the significant variabilities between the sample group’s mean comparisons (*p* < 0.05). Statistical measurements were performed using Microsoft Excel 2016 and Origin 2018. BBD data were processed by Design-Except 8.0.6 software.

## 4. Conclusions

This investigation provides a novel approach to producing a new nutritious and functional beverage using probiotic fermentation. The results showed that apple–tomato pulp could act as a matrix for probiotic fermentation, while increasing the viable cell count, organic acids, and antioxidant activity.

Quadratic polynomial models could well predict and describe the results of the viable cell count and sensory evaluation of fermented apple–tomato pulp. The optimum apple–tomato pulp fermentation conditions were determined by BBD as inoculum size = 6.5%, temperature = 34.5 °C, and apple: tomato ratio = 1:1. Under the experimental conditions, the viable cell count reached 9.02 lg(CFU/mL), and the sensory evaluation score was 32.50.

The physicochemical properties, antioxidant capacity, and volatile compounds of the optimized samples were evaluated. Fermentation reduced the pH and total sugar, and reducing sugar, while significantly increasing the total polyphenols, flavonoids, and antioxidant capacity of apple–tomato pulp. In comparison with the unfermented apple–tomato pulp, the TPC, TFC, DPPH, ABTS, and FRAP increased in fermented pulp by 21.28%, 22.22%, 40.91%, 22.60%, and 3.65%, respectively, indicating that fermentation effectively improves the polyphenol and flavone content and antioxidant activity of apple–tomato pulp. Moreover, fermentation by LAB had a great impact on the types and concentrations of volatile metabolites in apple–tomato pulp. A total of 55 volatile flavour compounds were detected among the uninoculated samples and fermented samples before and after fermentation. Alcohols, esters, and acids were the main contributors to the aroma.

## Figures and Tables

**Table 1:** Box–Behnken experimental design in the original and experimental results.

Run	Independent Variables (Actual and Coded Values)			Response	
	Inoculum Size (*X*_1_ %)	Temperature (*X*_2_ °C)	Apple: Tomato Ratio (X_3_ *V*:*V*)	Viable Cell Count (*Y*_1_ lg(CFU/mL))	Sensory Evaluation (*Y*_2_ Scores)
1	6(0)	34(0)	1:1(0)	7.87	27.68
2	6(0)	31(−1)	1:2(1)	8.05	30.12
3	6(0)	34(0)	1:1(0)	8.35	28.82
4	6(0)	37(1)	1:2(1)	9.24	28.01
5	8(1)	37(1)	1:1(0)	8.24	30.49
6	6(0)	31(−1)	2:1(−1)	8.79	31.87
7	4(−1)	34(0)	1:2(1)	8.49	29.80
8	4(−1)	37(1)	1:1(0)	9.03	29.31
9	8(1)	34(0)	1:2(1)	8.26	30.12
10	6(0)	34(0)	1:1(0)	8.51	29.74
11	8(1)	31(−1)	1:1(0)	8.11	29.52
12	6(0)	34(0)	1:1(0)	9.06	28.58
13	6(0)	34(0)	1:1(0)	9.00	33.90
14	8(1)	34(0)	2:1(−1)	9.01	34.14
15	6(0)	37(1)	2:1(−1)	8.89	34.56
16	4(−1)	34(0)	2:1(−1)	8.95	33.98
17	4(−1)	31(−1)	1:1(0)	9.06	33.69

**Table 2:** Variance analysis of regression model.

Source	Y_1_ Viable Cell Count, lg(CFU/mL)					Y_2_ Sensory Evaluation, Scores				
	Sum of Squares	*df*	Mean Square	*F* Value	*p* Value	Sum of Squares	*df*	Mean Square	*F* Value	*p* Value
Model	2.92	9	0.32	50.02	<0.0001	87.07	9	9.67	86.11	<0.0001
Linear										
*X*_1_	0.58	1	0.58	90.01	<0.0001	0.79	1	0.79	7.07	0.0326
*X*_2_	1.03	1	1.03	158.91	<0.0001	0.68	1	0.66	5.83	0.0464
*X*_3_ Interactions	0.099	1	0.099	15.28	0.0058	3.14	1	3.14	27.93	0.0011
*X_I_X*_2_	0.13	1	0.13	19.45	0.0031	2.64	1	2.64	23.51	0.0019
*X*_1_ *X*_3_	2.50 × 10^−5^	1	2.50 × 10^−5^	3.858 × 10^−3^	0.9522	0.87	1	0.87	7.78	0.0269
*X*_2_ *X*_3_	0.12	1	0.12	18.91	0.0034	0.078	1	0.078	0.70	0.4311
Quadratic										
*X*_1_^2^	0.22	1	0.22	33.19	0.0007	21.50	1	21.50	191.35	<0.0001
*X*_2_^2^	0.60	1	0.60	93.10	<0.0001	41.43	1	41.43	368.83	<0.0001
*X*_3_^2^	0.059	1	0.059	9.13	0.0194	8.57	1	8.57	76.32	<0.0001
Residual	0.045	7	6.479 × 10^−3^			0.79	7	0.11		
Lack of Fit	0.029	3	9.558 × 10^−3^	2.29	0.2200	0.36	3	0.12	1.13	0.4361
Pure Error	0.017	4	4.170 × 10^−3^			0.43	4	0.11		
R^2^	0.9847					0.9910				
Adj. R^2^	0.9650					0.9795				
Pred. R^2^	0.8363					0.9266				

**Table 3:** Pearson’s correlation coefficients between active substances and antioxidant capacity.

	TPC	TFC	DPPH	ABTS	FRAP
TPC	1				
TFC	0.96 *	1			
DPPH	0.90 *	0.98 *	1		
FRAP	0.97 *	0.98 *	0.94 *	1	
ABTS	0.95 *	0.94 *	0.87 *	0.95 *	1

*, Significant at *p* < 0.05.

**Table 4:** Composition and content of volatile compounds in unfermented and fermented apple–tomato pulp.

NO.	Compounds	CAS	Concentration/(μg/L)	
			Unfermented	Fermented
Alcohols				
1	1-hexanol	111-27-3	--	189.58
2	2-ethyl-1-hexanol	104-76-7	50.17	59.4
3	phenylethyl alcohol	60-12-8	11.56	24.88
4	ethanol	64-17-5	45.39	18.11
5	2-methyl-1-butanol	137-32-6	17.91	30.05
6	(E)-2-hexen-1-ol	928-95-0	--	25.29
7	6-methyl-5-hepten-2-ol	1569-60-4	23.92	37.57
8	(Z)-3-hexen-1-ol	928-96-1	--	23.09
9	linalool	78-70-6	6.12	11.7
10	geraniol	106-24-1	--	9.38
11	1-butanol	71-36-3	4.21	6.54
12	2-heptanol	110-43-0	2.95	3.87
13	linalool oxide	1365-19-1	1.72	3.48
14	2-methyl-3-octanol	26533-34-6	--	3.01
15	(S)-à,à,4-trimethyl-3-cyclohexene-1-methanol	10482-56-1	1.26	2.98
16	1-heptanol	111-70-6	--	2.31
17	1-octanol	111-87-5	--	2.1
18	1-pentanol	71-41-0	1.14	1.79
19	1-dodecanol	112-53-8	--	1.45
Total			166.35	456.58
Esters				
20	ethyl acetate	141-78-6	--	31.27
21	acetic acid-butyl ester	123-86-4	--	11.03
22	methyl butyrate	623-42-7	9.09	9.94
23	dibutyl phthalate	84-74-2	5.83	9.62
24	acetic acid-hexyl ester	142-92-7	17.46	2.93
25	1-butanol-2-methyl-acetate	624-41-9	25.16	4.48
26	2-hexanol-acetate	5953-49-1	--	1.42
27	formic acid-heptyl ester	112-23-2	--	2.31
28	formic acid-octyl ester	112-32-3	--	2.1
29	butanoic acid-3-hydroxy-butyl ester	53605-94-0	--	1.42
30	2,2,4-trimethyl-1,3-pentanediol diisobutyrate	6846-50-0	--	5.19
Total			57.54	81.71
Acids				
31	Acetic acid	64-19-7	2.59	35.58
32	Nonanoic acid	112-05-0	1.63	5.74
33	Decanoic acid	334-48-5	--	2.56
34	2-ethyl-Hexanoic acid	149-57-5	--	1.78
35	(E)-2-Hexenoic acid	13419-69-7	2.65	1.57
36	Octanoic acid	124-07-2	2.57	11.62
Total			9.44	58.85
Ketons				
37	6-methyl-5-hepten-2-one	110-93-0	--	2.85
38	(E)-1-(2,6,6-trimethyl-1,3-cyclohexadien-1-yl)-2-buten-1-one	23726-93-4	3.07	--
39	2-undecanone	112-12-9	--	1.74
40	2-heptanone	110-43-0	2.21	3.04
41	3-octanone	106-68-3	1.54	--
42	neryl acetone	3879-26-3	2.95	--
43	á-damascenone	23726-93-4	3.07	9.41
44	trans-á-Ionone	79-77-6	1.46	--
Total			14.30	17.04
Aldehydes				
45	2-Hexenal	6728-26-3	15.25	45.82
46	Hexanal	66-25-1	11.89	--
Total			27.14	45.82
Phenols				
47	4-ethyl-Phenol	831-82-3	--	24.02
48	2-methoxy-Phenol	90-05-1	1.30	2.33
49	2-Methoxy-4-vinylphenol	7786-61-0	--	1.66
Total			1.30	28.01
Others				
50	2,3-dihydro-Benzofuran	496-16-2	1.21	60.96
51	4-Vinylphenol	2628-17-3	2.53	40.34
52	2-Isobutylthiazole	18640-74-9	1.82	1.42
53	(2-nitroethyl)-Benzene	6125-24-2	2.33	3.30
54	4-nitrophthalamide	13138-53-9	1.52	--
55	dodecamethyl-Cyclohexasiloxane	540-97-6	--	1.55
Total			9.41	107.57

## Data Availability

Data will be made available upon request from the corresponding authors.

## Sample Availability

Samples of the compounds are not available from the authors.
